# The effects of gases from food waste on human health: A systematic review

**DOI:** 10.1371/journal.pone.0300801

**Published:** 2024-03-27

**Authors:** Paulina Rudziak, Evans Batung, Isaac Luginaah

**Affiliations:** 1 Department of Schulich School of Medicine and Dentistry, Western University, London, ON, Canada; 2 Department of Health Sciences, Western University, London, ON, Canada; 3 Department of Geography and Environment, Western University, London, ON, Canada; University of Birmingham, UNITED KINGDOM

## Abstract

Food waste is a routine and increasingly growing global concern that has drawn significant attention from policymakers, climate change activists and health practitioners. Amid the plurality of discourses on food waste-health linkages, however, the health risks from food waste induced emissions have remained under explored. This lack of evidence is partly because of the lack of complete understanding of the effects of food waste emissions from household food waste on human health either directly through physiological mechanisms or indirectly through environmental exposure effects. Thus, this systematic review contributes to the literature by synthesizing available evidence to highlight gaps and offers a comprehensive baseline inventory of food waste emissions and their associated impacts on human health to support public health decision-making. Four database searches: Web of Science, OVID(Medline), EMBASE, and Scopus, were searched from inception to 3 May 2023. Pairs of reviewers screened 2189 potentially eligible studies that addressed food waste emissions from consumers and how the emissions related to human health. Following PRISMA guidelines, 26 articles were eligible for data extraction for the systematic review. Findings indicate that emissions from food waste, such as hydrogen sulphide, ammonia, and volatile organic carbons, can affect human endocrine, respiratory, nervous, and olfactory systems. The severity of the human health effects depends on the gaseous concentration, but range from mild lung irritation to cancer and death. This study recommends emission capture technologies, food diversion programs, and biogas technologies to reduce food waste emissions.

## Introduction

Food waste is a common global issue–the latest Food and Agricultural Organization (FAO) report on food waste approximates 1.3 billion tonnes of food in the world is lost or wasted each year [1]. Food waste emits harmful gases, such as CO_2_, H_2_S, CH_4_, N_2_O, and PM_2.5_, that are detrimental to human health [2–4]. Emissions from food waste can negatively impact human health directly and indirectly. These impacts may include an increasing number of respiratory issues, and mild and severe headaches [2, 5]. The human health impacts have led to many global emission-related food waste policies and goals. For example, the FAO plans to halve the per capita global food waste at the retail and consumer levels in response to Sustainable Development Goal 12− Responsible Consumption and Production by 2030 [1]. In this study, composting, a form of food waste, is used to measure and characterize food waste emissions [2, 6].

### Level of analysis

For this systematic review, food is defined as edible products that contribute to human nourishment [7]. The definition of food waste will be “discarded food” from households, restaurants, and food catering services. Kitchen food waste mainly consists of fruit, vegetables, meat and bones, bread, fish bones, pasta, shellfish, rice, eggshells, coffee grounds, and dairy products [8]. Residential food waste can quickly decompose and produce odours because of its nature, high organic content, chemical mixtures, and sensitivity to room temperature [6].

Although food waste occurs in multiple stages of the food chain, focusing on the consumer level is important because of the relationship between consumption and food waste reinforced by the rapid industrialization, urbanization and economic development that have increased food waste levels [9]. Food waste estimates show that anthropogenic influence accounts for nearly one pound of wasted food per person per day [10, 11]. Wasted food is equivalent to over 30 million acres of cropland each year, mainly accounting for grains, oilseeds, vegetables, fruit, and dairy [10]. Fruits and vegetables are among the most wasted products [11]. Restaurants are guilty of serving portions too large for people to gauge higher price and profit margins. As a result, lots of food is wasted if the consumer does not like the idea of eating leftovers the next day [12]. Moreover, hotels, convention centres, resorts, and banquet halls that host large gatherings throw away a lot of viable food [13].

Metaphorically, if food waste were a country, it would be the third-largest greenhouse gas emitting country [1]. Thus, research on food waste emissions from households and consumer outlets needs to be the prime area of focus for their direct and indirect impacts on human health. Furthermore, most studies addressing food waste emissions are in contexts that vary in climatic conditions; thus, differences between food waste emissions will be assessed to account for different climatic conditions of composting areas. Warmer months with low aeration provide optimal conditions for food waste to emit gases at high concentrations [9]. Hence, food waste gas concentrations will be discussed in relation to odour thresholds (maximum tolerable gaseous concentration without odour annoyance) and olfactometry thresholds (the ability to change olfactory cell physiology) [9, 14].

### The current solutions to food waste emissions

Alternatives to food waste emissions have been explored, such as generating biofuels to power vehicles, heat homes, and generate electricity [15]. In this manner, food waste emissions are reused rather than released into the atmosphere which can have deleterious effects due to imbalances. Biofilters for composting sites have been experimented with to understand which filtering method best limits food waste emissions [14, 16]. In addition, food diversion systems have also been explored to ship untouched food from restaurants and catering events to local food banks and provide nourishment to people in need of food [13]. Despite these innovations, the net effect of the unprocessed food waste is negative as emissions continue to harm human health. It is therefore unsurprising that alternative methods to releasing food waste emissions into ambient air are being intensively explored [16].

### Relevance of this study

Previous systemic reviews addressing food waste have focussed on production and agricultural food waste, and loss prevention and mitigation techniques, but less so on human health outcomes and consumer food waste emissions [17–20]. This systematic review is unique in that it combines the studies reporting on various foods that emit toxic emissions from a consumer level and studies focusing more on the human consequences of these gaseous emissions. Such a review illustrates a clear link of food waste emissions and the impact on human health.

### Hypothesis and objectives

For this review, we hypothesize that food waste emissions will pose respiratory, nasal, and social health issues based on the current food waste evidence in the literature. Accordingly, the objective of this study is to review analyses of how gas emissions from food waste can impact human health both directly and indirectly. The objective will be accomplished by summarizing findings from literature and creating an inventory of food waste gases and their associated health effects. In this systematic review, direct human health impacts relate to physiological effects on the human body. Indirect human health effects relate to secondary outcomes, such as fresh-water acidification, which can lead to human health effects.

## Methods

We began the design by searching the international database of Prospectively Registered Systematic Reviews (PROSPERO) in “Health” and “Social care” for similar or identical reviews prior to study commencement, and none were found [21]. The Preferred Reporting Items for Systematic Reviews and Meta-Analyses (PRISMA) guidelines were then employed to systematically identify and assess approaches to ensure consistent methods and analyses for this review [22]. To inform this systematic review, the databases Web of Science, OVID(Medline), EMBASE, and Scopus were searched from inception to 3 May 2023. These databases were selected based on their relevance to environmental and human health research. No restrictions on language or publication date were set in the databases. Three strings were searched in each database. The strings included keywords ‘food waste’ AND ‘human health’ AND ‘gases,’ and synonyms for gases were also searched, including ‘emissions’ and ‘vapours.’ An additional search strategy was later added to review compost emissions more broadly. The key words ‘compost emissions’ AND ‘human health’ were searched in Web of Science, OVID(Medline), EMBASE, and Scopus (S1 Fig).

**Fig 1:**
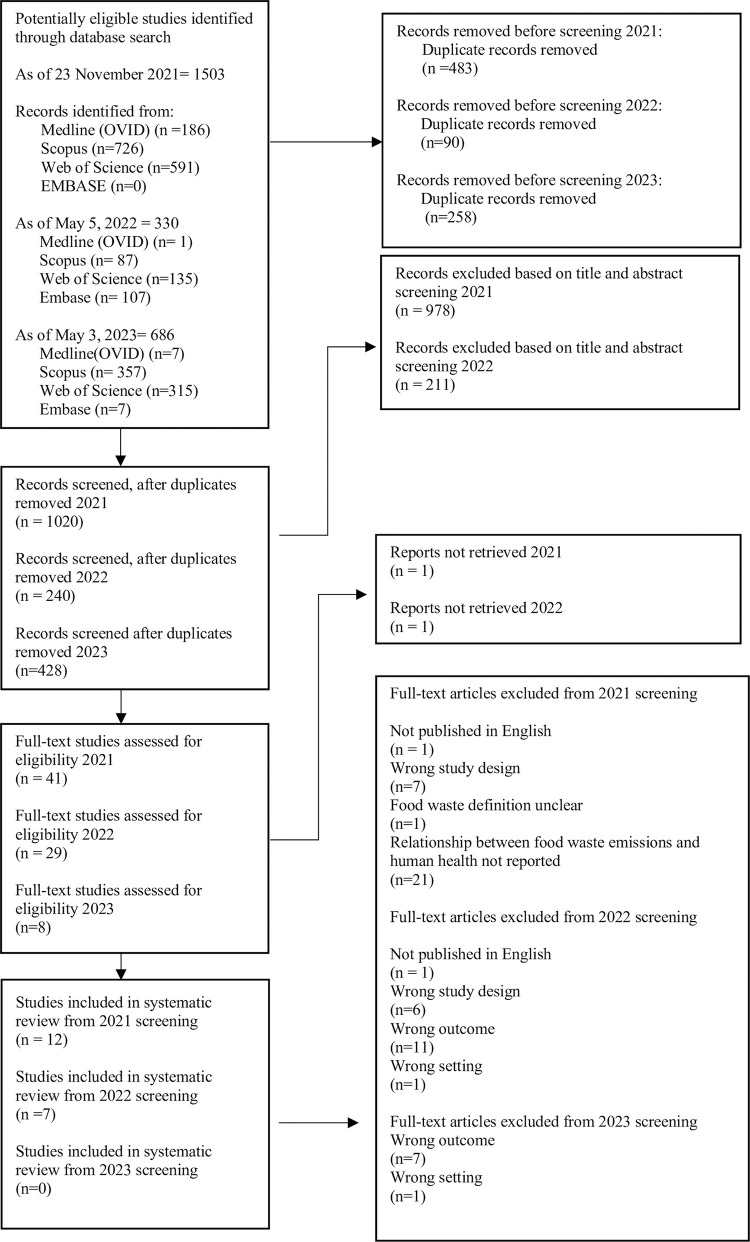
PRISMA flow-chart of study selection process.

For the first search strategy, a pair of reviewers screened 1020 potentially eligible titles and abstracts in the systematic review software Rayyan, after removing 483 duplicates (Fig 1) [23]. This software allows for uploads of search strategies from databases into a project folder, detects duplicates among the added titles and abstracts, and allows for highlights of keywords to help each reviewer with the inclusion of articles.

For the second and third search strategy, a pair of reviewers screened 668 potentially eligible titles and abstracts for the systematic review in the systematic review software Covidence, after removing 348 duplicates [24]. Covidence was used for the second search strategy because of the upgrades that were made available to facilitate the screening process with relative ease. Following training, pairs of reviewers independently screened all titles and abstracts, followed by full-text articles that were identified as potentially eligible. When necessary, a consensus was reached through a discussion.

The inclusion criteria for titles and abstracts consisted of primary research related to food waste emissions from the human consumer level, such as household food waste, restaurant food waste, and purposeful viable food discards from food retailers. Articles that report a mix of municipal solid waste, everyday garbage consisting of everything and anything, for food waste emissions data were not considered for the review [25]. This is because emissions from unsorted municipal solid waste cannot be generalized to the food waste within. The articles of focus are studies that report emissions from consumer food waste/ scraps and organic compost. Food waste from production, manufacturing, and commercial farming was also not considered because of differences in definitions of food waste across studies and the vast complexities involved. Title and abstracts also had to mention human health in context to be included for full-text screening.

A total of 70 (6.8%) articles were marked as discrepancies between reviewers after title and abstract screening and were resolved after discussion. To facilitate full-text screening, articles were restricted to the English language [26]. After title and abstract screening, 80 articles were eligible for full-text screening, and two articles were excluded because they did not have the full-text published. In total, 78 full-text articles from the search strategy itself were screened by a pair of reviewers. An excel sheet was formulated for full-text screening with identical inclusion criteria (S2 and S3 Figs). Criteria for inclusion consisted of primary research articles that addressed food waste, waste emissions, human health, and articles published in the English language. The link to human health could be direct or indirect (measured or mentioned in context). Editorials, commentaries, and reviews were excluded to focus on evidence-based primary research. After resolving 13 (32.5%) discrepancies, 19 out of 78 articles were included for data analyses after full-text screening, and 59 out of 78 articles were excluded (Fig 1). The excluded articles lacked addressing human health directly or indirectly and consisted of varying definitions of food waste that were too challenging too extract. The bibliography of included articles was searched for relevance to minimize the risk of not including relevant studies. Seven articles were included from searching citations of included articles, expanding the included full-text articles for data analysis to 26 articles (S4 Fig).

To evaluate the quality of each publication selected for the systematic review, a modified version of the “McMaster University’s Critical Review Form” was used to thoroughly critique the studies (S5 and S6 Figs) [27]. Reviewers individually used the modified form to critique articles in the following areas:

- purpose of the study,
- background information,
- study design,
- outcomes,
- interventions,
- results, and
- conclusions of article.

The Joanna Briggs Institute critical appraisal form for case reports was used for the one included case report [28]. To facilitate the appraisal process, excel files were created using the checklists of the critical appraisal reports. Critical appraisals were completed in duplicate, and opinions on the credibility of articles were discussed and decided amongst the reviewers. To facilitate the write-up process, annotations of included full-text articles were created. Included in the annotations were methodologies, results, limitations, and recommendations. The annotations helped to determine common themes among included articles to help articulate the systematic review and helped determine the set-up of the results summary table. The summarization of ideas in articles is subjective to a certain degree but allows researchers to compare views and extract shared meanings [29].

## Results and discussion

### Overview

**Fig 2:**
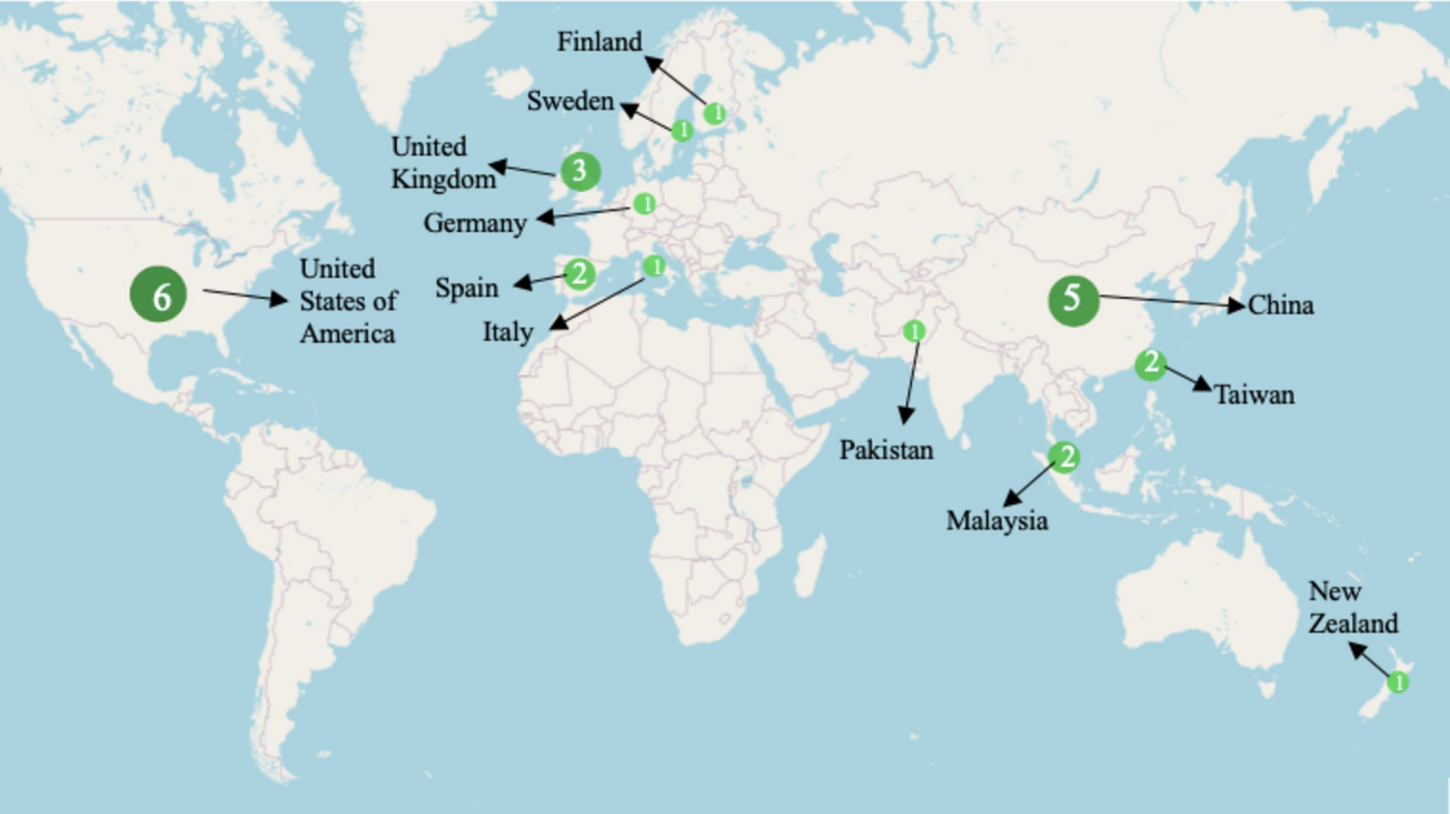
Country Representation of Included studies. The python-generated map includes circles of various sizes representing the number of studies included from various countries. China, Europe, and the United States of America were the main origins of studies conducted. Most studies were conducted in Asia.

**Fig 3:**
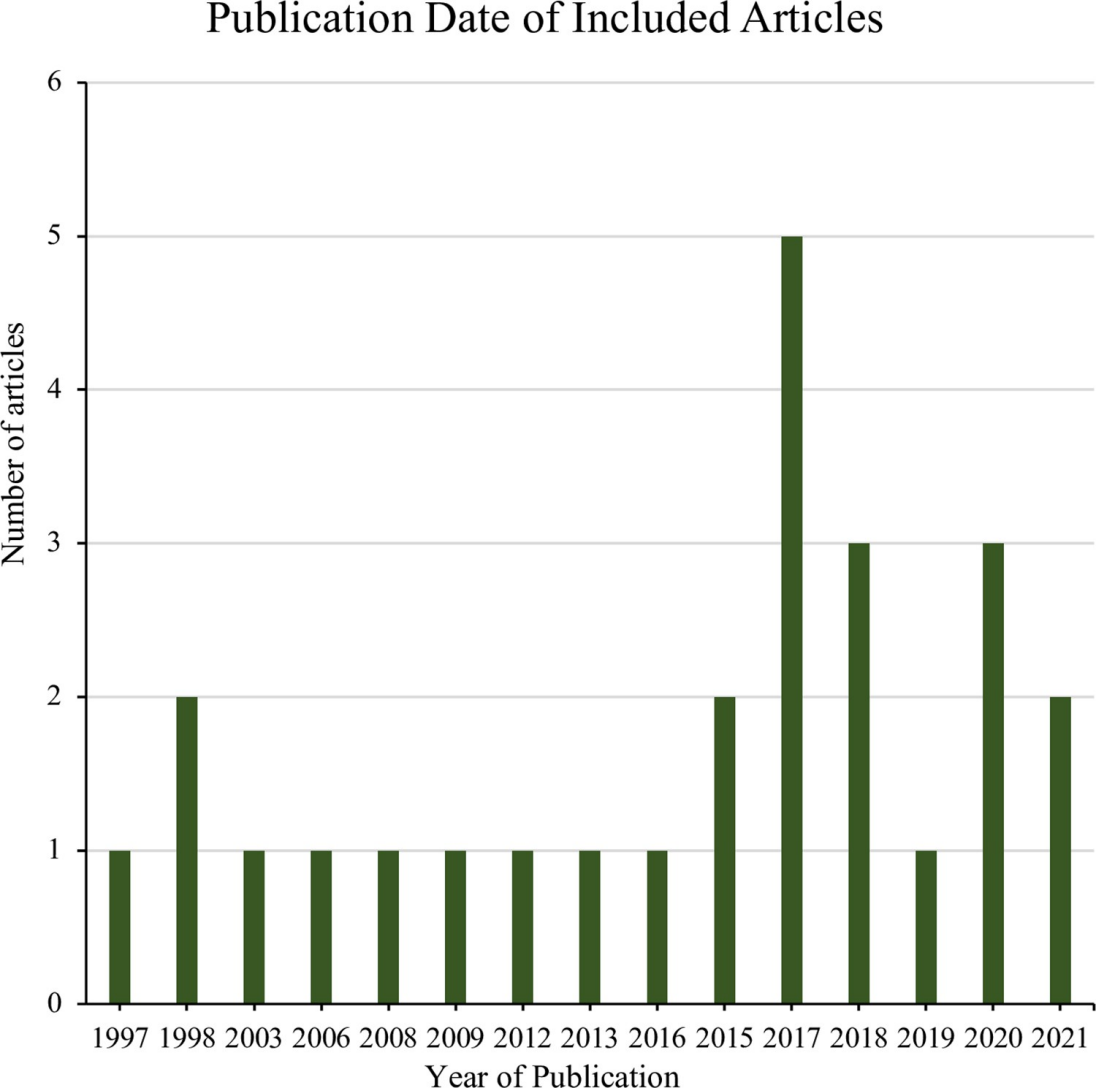
Publication dates of included studies. The year 2017 had the most frequent publication date, and the last decade showed the most publications in general.

Studies analyzed research in various countries with different food waste emission regulations. Olfactory and odour thresholds differed among countries, likely because of the change in climate and governmental stipulations [4, 9, 14]. The 26 included studies were conducted mainly in Asia (38%), Europe (35%), and the United States of America (23%) (Fig 2). Most of the articles were also published within the last decade (65%) (Fig 3). The number of articles reporting mild to severe direct human health impacts is summarized in Table 1. Because of the variability in composition, temperature, climate, and moisture of food waste across countries, the summary of food waste emissions in Table 2 is not generalizable for the entire world, but the impact on human health from such gases is universal.

**Table 1:** Human health impacts of food waste emissions.

Direct human health impacts	Number of articles (%)
Lung irritation	4 (17%)
Olfactory cell changes	5 (22%)
Decreased respiration	2 (9%)
Endocrine, cardiovascular, and nervous system complications	3 (13%)
Carcinogen	3 (13%)
Loss of consciousness and death	2 (9%)
High toxicity	2 (9%)

**Table 2:** Summary of food waste emissions and their impacts on human health.

Emissions (gaseous phase)	Gas quantities	Non-threatening level (mg/m^3^)	Specific source of emission from food	Source of emission during compost	Odor threshold	Indirect human health impact	Direct human health impact	Reference(s)
Methane	(0.77–2.5kg/ tonne fresh matter)	<1.0E-06	Organic matter^b^	Biodegradation, and anaerobic digestion	NR^a^	Global warming	NR^a^	[30, 31]
Carbon dioxide	15.25 kg CO_2_ eq, 88 kg CO2 eq	NR^a^	Organic matter^b^	Biodegradation	NR^a^	Global warming	NR^a^	[30, 32]
Ammonia	(0.05–3.9kg/ tonne fresh matter), 800ppb, (0.15–0.5mg/m^3^), (867–17347 μg/m^3^)		Organic matter^b^	Microbial decomposition, and anaerobic digestion	1.0629mg/m^3^	Soil and water-body acidification	Lung irritation, changes to sensory receptors of olfactory cells, and inflammation of olfactory tissues	[4, 9, 14, 30]
Acetic acid	(612μg/m^3^)	<1.0E-06	Fruits	Fermentation	NR^a^	Acidification	Lung irritation, and olfactory cell changes	[14, 31]
Nitrous oxide	(0.05–1.8kg/ tonne fresh matter)	<1.0E-06	Organic matter^b^	Combustion	NR^a^	Smog, tropospheric ozone precursor	Lung irritation	[2, 30, 31]
Sulphur dioxide	86.58 kg SO_2_ eq, (0.18–1.2397mg/m^3^)	<1.0E-06	Fermented foods, broccoli, cabbage, kale	Combustion	NR^a^	Soil and water-body acidification, smog, and tropospheric ozone precursor	Lung irritation	[2, 16, 30, 31]
Carbon monoxide	NR ^a^	NR^a^	Organic matter^b^	Aerobic decomposition, and combustion	NR^a^	NR^a^	Decreased respiration	[2]
Hydrogen sulphide	2.9mg/m^3^,	<1.0E-06	Fish	Organic matter decomposition, conversion to biogas, sorting, and anaerobic fermentation	NR^a^	Acid rain	Death, loss of consciousness, high toxicity, disruption of electron transport chain, decreased respiration, neurological deficits, paralysation of olfactory receptors, seizures, headaches, vertigo, coma, photophobia, blurring of vision, cardiac arrest, ischemia, and shut down of the central nervous system	[2, 3, 9, 31]
Terpenes	1.5μg/m^3^, (0.009–1.3764mg/m^3^), (29–20580μg/kg)	<1.0E-06	Citrus foods, pulp, oils, mixed nuts, cheese, and ground beef	Aerobic or anaerobic digestion	(12–3890μg/m^3^), 83μg/m^3^	Ne^r^^a^	Lung irritant	[9, 16, 31, 33, 34]
Alcohols	5.9x10^2^μg/m^3^, (0.2132–20.366mg/m^3^), (43.90–85.31mg/m^3^), (28–1631μg/kg)	<1.0E-06	Lipids and proteins	Anaerobic digestion	997μg/m^3^	NR^a^	Non-carcinogenic irritant	[8, 9, 16, 31, 33–36]
Aromatic compounds	10.2μg/m^3^, (27–2302μg/kg)	<1.0E-06	Roasted, fried, and smoked foods, mixed nuts, ground beef, pork bacon, raw banana, canned tuna, cake with icing, peanut butter, cookies, olive oil, pizza, bologna, and potato chips	Anaerobic digestion	1265μg/m^3^	NR^a^	Respiratory, endocrine, and nervous system complications	[9, 31, 33, 34, 37]
Halogenated compounds	20.6μg/m^3^, (0.056–1.433mg/m^3^), (2.09–2.11mg/m^3^)	<1.0E-06	Fish, eggs, chicken, beef	Anaerobic digestion	3048μg/m^3^	NR^a^	Possible carcinogen to humans	[16, 31, 33, 38]
Toluene	(0.0158–0.6510mg/m^3^), (11–64μg/m^3^), (1.1E-04–2.7E-04 mg/m^3^), 55μg/kg	<1.0E-06	Mixed nuts, cheese, ground beef, doughnuts, butter, margarine	Hydrothermal hydrolysis	5888μg/m^3^	NR^a^	Irritation and discomfort in olfactory cells	[9, 14, 16, 34, 37]
Methanethiol	(0.0048–0.8836mg/m^3^)	<1.0E-06	Broccoli, cabbage, kale	Degradation, treatment process	NR	NR^a^	Irritation and discomfort in olfactory cells	[9, 31]
Dimethyl sulphide	(0.0292–0.2855mg/m^3^), (759μg/m^3^), 37μg/kg	<1.0E-06	Broccoli, cabbage, kale	Degradation	NR	NR^a^	Irritation and discomfort in olfactory cells	[9, 14, 31]
Dimethyl disulphide	(0.0133–2.3886 mg/m^3^)	<1.0E-06	Broccoli, cabbage, kale	Degradation	NR	NR^a^	Irritation and discomfort in olfactory cells	[9, 31, 34]
Acetaldehyde	(0.0433–3.1658mg/m^3^), (28–1650μg/kg)	<1.0E-06	Organic matter^b^	Pre-treatment and treatment processes	2.75μg/m^3^	NR^a^	Irritation and discomfort in olfactory cells	[9, 31, 33]
Isobutane	(0.0072–3.4271mg/m^3^), (0.651–4.429mg/m^3^)	<1.0E-06	Organic matter^b^	Fermentation	NR	NR^a^	Non-carcinogenic irritant	[9, 16, 31]
Pentane	(0.0017–1.5188mg/m^3^), (0.651–4.429mg/m^3^)	<1.0E-06	Organic matter^b^	Fermentation	NR	NR^a^	Non-carcinogenic irritant	[9, 16, 31]
Butane	(0.0092–3.6602mg/m^3^), (0.651–4.429mg/m^3^)	<1.0E-06	Organic matter^b^	Fermentation	NR	NR^a^	Non-carcinogenic irritant	[9, 16, 31]
Naphthalene	(0.0022–0.0057mg/m^3^), (1.E-05–3.07mg/m^3^)	<1.0E-06	Organic matter^b^	Fermentation	NR	NR^a^	Possible carcinogen, hazardous	[9, 16, 31]
1,2,4-Trimethylbenzene	(5.7E-02- 0.80mg/m^3^)	<1.0E-06		Fermentation	NR	NR^a^	Possible carcinogen, hazardous	[16, 31, 37]

NR^a^ = not reported

^b^Organic matter = specific food constituents not reported

### Classification of food waste emissions

Typical for composting conditions, food waste was mixed with a percentage of wood chips, usually 30% in included studies [39]. A typical food waste composting process includes sorting/crushing food mechanically, hydrothermal hydrolysis, then conversion into a liquid for anaerobic digestion or conversion into a solid for aerobic fermentation [31]. During all stages of the composting process food waste emissions can be released in various levels of toxicity into the surrounding environment [31]. Composting conditions across included studies were not variable enough to emit higher amounts of toxic gases, but higher concentrations of food waste gases were typically collected in winter months [9, 31]. Most studies used a gas chromatography-mass spectrometer to identify the emissions from food waste [4, 9, 14, 31]. Emissions from food waste during various stages of composting were compared to odour thresholds recommended by environmental protection agencies of the countries represented in the studies [14].

The most common harmful gases captured from food waste include some volatile organic carbons (VOCs) (such as terpenes and sulphur compounds); 1,2,4-trimethylbenzne; aromatic compounds and hydrogen sulphide because of their associated human health effects [9, 14, 16]. Food waste composting is influenced by the concentration of emissions, which is dependent on aeration, temperature, moisture, and storage [34]. Food waste decomposes more at higher temperatures, and odour intensity increases linearly with increasing storage time and increasing temperatures [6]. The measure of total organic carbon can be used to quantify the amount of organic matter present within compost [6]. Quantification of organic matter is important to determine the homogeneity of the compost, and if different factors contribute to high emission concentrations or high odour intensity levels.

Odour intensity can be measured by two evaluators smelling buckets of food waste at 1, 4, 7, 10, and 14 days apart, and at 20°C and 8°C temperature differences [6]. Generally, the more extended storage periods at 7, 10, and 14 days at 20°C had a scaled odour intensity described as overpowering, intolerable for any length of time, and acute exposure could change olfactory physiology. The odours in common with all the buckets were ammonia and isovaleric acid. Both have low odour thresholds and strong scents, possibly causing the strong odours from all the buckets evaluated at 20°C. At 8°C, the emissions were considered very distinguishable, irritable, and objectionable [6]. The most common harmful gases captured from food waste include some volatile organic carbons (VOCs) (such as terpenes and sulphur compounds); 1,2,4-trimethylbenzne; aromatic compounds; and hydrogen sulphide that tested above their designated odour thresholds [9, 14, 16]. Hundreds of VOCs emitted from compost, and they pose a hazard to human health [34]. The VOCs shown on Table 2 mainly include terpenes, butane, halogenated compounds, aromatic compounds, isobutane, pentane, butane, dimethyl sulphide, and dimethyl disulphide [16, 31, 34, 40]. VOCs emit odours during conditions of low aeration and high moisture levels, increasing the volatility of compounds by increasing their vapour pressure and availability of microorganisms to degrade the food waste [34]. Given the various health effects described in Table 2, VOC emissions variably break down because of the uneven distribution of methanogenic bacteria used to break down composted food and varying molecular weights of compounds [34, 40]. The VOCs with lower molecular weights can quickly enter the gaseous phase and become volatile [41]. In the aqueous compost phase, VOCs can solubilize easily under high temperatures and are emitted at high rates into the surrounding atmosphere [34, 40]. Concentrations of VOCs are higher and more concerning for human health in indoor settings than in outdoor settings [16]. Human health is compromised from long-term VOC emissions from food waste because of the carcinogenicity of VOCs, their ability to irritate olfactory cells, change their physiology, and compromise endocrine, respiratory, and nervous systems [31].

### Seasonal fluctuations in food waste emissions

Dietary changes in the summer months create food waste mainly consisting of fruits and vegetables, compared to high fat and protein foods consumed in the winter months [9]. Fruits and vegetables contain large amounts of sugars and carbohydrates that are easily converted into oxygenated organic compounds such as acetaldehyde. In January, people consume more eggs and meats that contain sulphur proteins, so sulphur malodors are common [9].

The difference between the measurements during the two seasons was higher water content and higher temperature during summer months, making decomposition easier [9, 34]. This difference is specific to China’s summer and winter months and follows Chinese odour regulations [9]. During September emission measurement collections, thresholds were exceeded for toluene (0.65173 mg m^-3^), dimethyl sulphide (0.00776 mg m^-3^), and acetaldehyde (0.00282 mg m^-3^) across multiple compost sites [9]. Ammonia emissions exceeded the threshold (1.0629 mg m^-3^) at one compost plant during September emission collections. During both September and January emission collections, thresholds were exceeded for methanethiol (0.00014 mg m^-3^), dimethyl disulphide (0.00862 mg m^-3^), and hydrogen sulphide (0.000581 mg m^-3^) across multiple compost sites. Hydrogen sulphide was the only gas that exceeded its threshold at every compost location in both September and January collections, suggesting its emission of a powerful odour [9]. A study completed in Taiwan compared food waste emissions during typically high temperature and humidity conditions of the compost plant to olfactory thresholds [14]. The emissions p-Cymene and ethylbenzene exceeded their olfactory thresholds (12 ug m^-3^) and (13 ug m^-3^), respectively, compromising the health of workers. Concentrations below the emission thresholds stated are considered safe for workers in the compost area [9, 14].

The emissions stated all had low odour thresholds, which means that even the slightest concentration above threshold levels can irritate workers and residents nearby, measured using an odour activity value (OAV). Hydrogen sulphide, dimethyl sulphide, dimethyl disulphide, methanethiol and acetaldehyde had large OAV values, causing great and sustained annoyance among workers and residents nearby. Large OAV values can detriment workers’ and residences’ health, well-being, and quality of life [9, 14]. Hydrogen sulphide is arguably the most lethal food waste emission, having the capability to cause cardiovascular, respiratory, neurological, and vision complications that can lead to impaired functioning and death [3]. Toluene has a low OAV value and poses minimal annoyance for workers and residents nearby [9].

### Connection to human health

The evidence provided here shows a positive linkage between food waste emissions and both direct and indirect human health impacts. Food waste can disrupt olfactory cell functioning, atmospherically spread to nearby residents, and cause occupational safety concerns [4]. One of the main concerns of food waste emissions is the possible impairment of olfactory cells [4, 9, 14]. Some studies used the odour thresholds of chemical compounds that varied among studies, to determine the hazard potential for workers at the compost site and residents living nearby [9, 14, 16]. Odours can irritate olfactory cells, which are vulnerable to impairment from strong odours. This can in turn, decrease the safety and quality of life for individuals affected [14, 41, 42]. For example, individuals may not be able to tell when food is spoiled by the smell and may ingest harmful products [4, 14]. In another example, the odours from Taiwanese food waste plants forced residents nearby to protest, causing a shutdown and an investigation of the plants [4]. Proper aeration and controlling temperatures of food decomposition are essential to lower concentrations of food waste emissions [16, 31].

Table 2 includes information about respiratory health issues related to food waste emissions. Respiratory health issues range from least severe, lung irritation, to most severe, carcinogenicity. The respiratory health reports came from complaints of compost plant workers and residents living nearby them [4, 9, 16]. The highly volatile gaseous compounds, such as VOCs, had the most detrimental respiratory human health effects [16].

Moreover, the emissions of food waste, such as ammonia gas, have social health costs in addition to environmental health and human physiological health. For example, ammonia is a precursor for the formation of Particulate Matter 2.5, which are ultra-fine particles known to cause severe respiration complications by lodging deep into alveoli, obstructing their function and shape [43]. Particulate matter can travel long distances, affecting populations not only in the nearby composting location [2]. Arguably, a possible solution to human exposure to food waste emissions is to locate composting sites in very sparsely populated areas [2]. However, an environmental inequity would arise because rural inhabitants would be exposed to polluted air from food they mostly did not throw away.

### Key components in food waste

In addition to gaseous chemical emissions, biological aerosols and endotoxins are emitted from food waste compost and pose potentially serious human health respiratory repercussions [44]. Such toxicity is especially harmful to humans because of the biological aspect that can create more severe human health complications [45].

### Bioaerosols

Bioaerosols are aerosols with any biological origin; bacteriological and fungal aerosols are mainly reported in this review [44]. The highest concentration of bioaerosols is found at the boundary areas for site collection, which may be due to accumulation from being carried upwind [45]. Peaks in bioaerosol emissions occur at the 100 and 150m boundary downwind of the composting sample, possibly because of the buoyancy effect, causing some bioaerosols to rise above sampling height until cooled to sink back to a sampling height [45]. Inhalation of bioaerosols pose respiratory challenges for humans, such as inducing allergies, sensitivity, and infectious disease [46]. A standard for microbes in the air is not universally settled, but not exceeding 1000cfu/m^3^ is recommended [39]. Size of bioaerosol particles matter since smaller particles can penetrate deeper into the respiratory system, making it more difficult to for lungs to recover [46]. Bioaerosols of particles >7.1μm reach the nasal cavity, 4.7–7.1μm reach the pharynx, 3.3–4.7μm reach the trachea and primary bronchi, 2.1–3.3μm reach the secondary bronchi, 1.1–2.1μm reach the terminal bronchi, and 0.65–1.1μm reach the alveoli [47]. Such deep penetrations can pose respiratory challenges as mentioned previously [46]. Bacteria emitted from a United Kingdom compost site were <0.6μm in diameter, threatening deep tissues of human lungs. Some larger particles were actinobacteria and firmicutes, with a diameter >3.3μm.

Fungal aerosols are also considered bioaerosols and they range in size. Common fungal aerosols found around the world in compost are *Penicillium*, *Aspergillus*, *Emericella*, and *Paecilomyes* [39, 48]. In some samples, such as a UK composting site, *Capnodiales* were found to have a diameter >3.3μm, and made up more than 25% of the fungal community in the compost. *Eurotiales* have a diameter between 4.7–1.1μm and made up more than 50% of the fungal community in the compost. *Ascomycota* have a diameter between 3.3–4.7um and make up a little more than 6% of the fungal community in the compost. *Glomeromycota* have a diameter between 1.1–0.65μm and make up 6% of the fungal community in the compost [47]. Table 3 classifies bioaerosols and gases by odour thresholds or diameter.

**Table 3:** Odour thresholds and diameters of food waste emissions pooled.

Emission	Emission Type	Odour threshold	Diameter (μm)	Composting type	Country	Author(s)
*Capnodiales*	Bioaerosol	NR	>3.3	Food waste standard	United Kingdom	[47]
*Eurotiales*	Bioaerosol	NR	1.1–4.7	Food waste standard	United Kingdom	[47]
*Ascomycota*	Bioaerosol	NR	3.3–4.7	Food waste standard	United Kingdom	[47]
*Glomeromycota*	Bioaerosol	NR	0.65–1.1	Food waste standard	United Kingdom	[47]
*Actinobacteria*	Bioaerosol	NR	>7–0.65	Food waste standard	United Kingdom	[47]
*Bacteroidia*	Bioaerosol	NR	>7.1–1.1	Food waste standard	United Kingdom	[47]
*Flavobacteriia*	Bioaerosol	NR	>7–0.65	Food waste standard	United Kingdom	[47]
*Sphingobacteria*	Bioaerosol	NR	>7–0.65	Food waste standard	United Kingdom	[47]
*Bacilli*	Bioaerosol	NR	>7–0.65	Food waste standard	United Kingdom	[47]
*Clostridia*	Bioaerosol	NR	>7–1.1	Food waste standard	United Kingdom	[47]
*Alphaproteobacteria*	Bioaerosol	NR	>7–0.65	Food waste standard	United Kingdom	[47]
*Betaproteobacteria*	Bioaerosol	NR	>7–2.1	Food waste standard	United Kingdom	[47]
*Gammaproteobacteria*	Bioaerosol	NR	>7–0.65	Food waste standard	United Kingdom	[47]
Acetic acid, methyl ester	Gas	0.7–63	NR	windrow	Finland	[39]
Acetic acid, ethyl ester	Gas	0.0196–655	NR	windrow	Finland	[39]
Propanoic acid	Gas	0.3–5	NR	windrow	Finland	[39]
Butanoic acid, methyl ester	Gas	0.005–0.077	NR	windrow	Finland	[39]
Butanoic acid, ethyl ester	Gas	0.13–0.28	NR	windrow	Finland	[39]
Acetic acid, butyl ester	Gas	0.03–1750	NR	windrow	Finland	[39]
Hexanoic acid, ethyl ester	Gas	0.0043–0.03	NR	windrow	Finland	[39]
Octanoic acid, ethyl ester	Gas	0.002–0.01	NR	windrow	Finland	[39]
Acetic acid, bornyl ester	Gas	0.44	NR	windrow	Finland	[39]
Dimethyl sulphide	Gas	3.0 ppb_v_	NR	Community composting	Italy	[49]
Dimethyl disulphide	Gas	2.2 ppb_v_	NR	Community composting	Italy	[49]
Alpha-Pinene	Gas	18 ppb_v_	NR	Community composting	Italy	[49]
Beta-Pinene	Gas	33 ppb_v_	NR	Community composting	Italy	[49]
p-Cymene	Gas	1200 ppb_v_	NR	Community composting	Italy	[49]
Limonene	Gas	38 ppb_v_	NR	Community composting	Italy	[49]
Ethyl isovalerate	Gas	0.013 ppb_v_	NR	Community composting	Italy	[49]

NR = not reported, raw data unavailable

### Endotoxins

Endotoxin emissions released from compost are potent and proinflammatory for human respiration; associated with ailments such as airway obstruction, pneumonitis, bronchitis and decreased lung function [50]. Endotoxins do not disperse into communities near compost sites as dispersal is maxed at 280m past the compost site. Thus, endotoxins mainly threaten the health of plant workers [51]. Endotoxin emission >50EU/m3 can impose damage to human respiration systems, very low emissions of endotoxins (~2.3EU/m3) were reported in the study, which cannot justify the respiratory complaints of the occupational workers [51]. Table 4 summarizes the range of colony forming units (CFUs) for fungal emissions and general endotoxin emissions from compost.

**Table 4:** Colony forming units of fungi and endotoxins.

Emission	Emission Types	CFU range (CFU/m^3^)	Author
Aspergillus fumigatus	Bioaerosol	5300–30000	[45, 51]
Actinomycetes	Bioaerosol	40000–10000	[45]
Endotoxins from gram-negative bacteria	Endotoxins	400000–900000	[45]

### Potential solutions to food waste emissions

Based on the findings, we propose potential solutions. Considering the health threats that food waste can pose to human health, we suggest several pathways in which this effect can be avoided. First, an eco-friendlier solution to uncompromised, ready-to-eat food is to partner with a food bank to provide ready meals and ingredients that would have otherwise been thrown away [13]. In about a year, a pilot study donated 24,703kg of recovered food, providing about 45,383 meals, which prevented the release of 82.8 MT CO_2_eE of CO_2_, 15.5 MT CO_2_E of CH_4_, and 8.5 MT CO_2_E of N_2_O [13]. Not only does redirecting food decrease landfill area use, but it also provides food to starving populations which can play a critical role in increasing the health and well-being of food insecure and malnourished groups.

Second, we also propose the use of organic food waste to create biofuel to power vehicles and provide electricity and heating [15]. Biofuel creation can still emit typical food waste gases, so a gas capture technology is necessary [2]. Biofuel for vehicle use could avoid climate change impacts caused by petrol fuel by capturing most food waste emissions and converting it into energy [15]. A study conducted in China measured the impact on human health from diverting food waste to create biofuel in Disability Adjusted Life Years (DALYs) [15]. The impact on human health from climate change and particulate matter was measured as 3.51x10^-6^ DALYs and 2.6 x 10^−4^ DALYs, respectively. Biogas used for electricity and heating had a human health damage of 10.65 x 10^−5^ DALYs/ tonne food waste. Biogas for city gas had a human health damage of 26.46 x10^-5^ DALYs/ tonne food waste. Biogas for vehicle use was calculated to have negative DALY values, considering the avoidance of petrol fuel [15].

A potential solution for food waste emissions from compost is to install a biofilter or bio-tricking filter to reduce gaseous food waste emissions, such as toxic hydrogen sulphide. A biofilter is a tower made from gravel, wood chips, food waste compost and coal bottom ash, whereas as a bio-trickling filter consists of a tower with polypropylene balls to filter material [2, 14, 16]. For example, a biofilter can capture 90% of ammonia emissions from composted food piles [14]. In general, less strong odorous gases, such as ammonia and VOCs, can be emitted at composting plants that use either biofilters or bio-trickling filters, because of deodorization [14].

### Public policies

Reduction of compost gas emissions would be advantageous to the physical and social health of workers and close neighborhoods [52]. Policies for diverting food waste can include a food redistribution program to redistribute edible food to food banks, and education. Educating the public and commercial food retail can include topics on food waste, portion size, food purchasing, planning and preparation, and enacting more served plated food rather than buffet style [13]. A redistribution program can target restaurants, banquet halls, convention centres, and catering services to collect viable food to donate to local food banks. Educating the public on planning meals before grocery shopping can limit food waste. In addition, educating the public on how to properly store certain foods can help decrease wastefulness. Educating commercial food retailers on how to preserve prepared food, waste less food during preparation, and serve smaller portion sizes. Transitioning from buffet-style food businesses to plated foods can significantly decrease food waste and decrease food preparation costs for the businesses. In this manner, public sectors and individuals can be educated on limiting food waste.

Encouraging cities to enact “green bin initiatives” with capture technologies can help decrease malodor complaints and associated human health consequences for compost plant workers and people residing near the plant [2]. In addition, capturing emissions could also create biofuels to generate electricity for nearby residents [15]. In this manner, people would be recycling food waste into a clean, usable energy source.

## Limitations and strengths

Notwithstanding our thorough search, only a few articles focused on consumer food waste, despite this sector growing in food waste gas emissions over the years as gross domestic product increases [13]. Furthermore, most of the articles in the search strategy that were excluded from this study focused on emissions during food production processes, rather than food waste processes. In addition, very few articles measured direct human health effects of food waste emissions, most focused on identification of gases and indirect human health links. This systematic review makes a crucial addition to the food waste literature because of the limited primary research and systematic reviews on consumer food waste and human health impacts, and calls upon researchers to explore more human impacts from food waste emissions.

A strength of this study is the duplication of abstract screening, full-text screening, and critical appraisals for all the search strategies that generated 1688 unique articles. PRISMA guidelines have also been thoroughly followed, increasing this article’s validity as a systematic review [22]. The critical appraisals of the included articles deemed each study to be conducted well, increasing the strength of the compiled data. Lastly, the compilation of data into Table 3 was a major accomplishment that strengthened the systematic review as a thorough synthesis of food waste emission impacts on human health.

## Areas for future research

Future primary research needs to address human health outcomes directly through clinical trials, like how a study [6] used two individuals to rate odours from buckets of food waste. Direct observations of human health outcomes can strengthen the current literature and provide clear evidence for relevant stakeholders to enact policies. In addition, a more holistic approach to human health effects needs to be studied to address the interconnectedness of environmental health and human health. Moreover, a study understanding the difference in food waste thresholds across countries could help address the worldwide human health risk of varying waste emission concentrations to workers and the public. Understanding the differences in odour thresholds by means of guidelines or standardization on documenting gas emissions and limits could warrant a meta-analysis in the food waste literature. Lastly, studies on global distillation could help address the worldwide effect of food waste emissions from individual countries and help implement international regulations.

## Conclusions

This study presents a compilation and categorization of evidence of food waste emissions and their impacts on human health. After analyzing 26 articles, this study found that food waste from consumers can emit gases, such as VOCs, ammonia, carbon dioxide, methane, and hydrogen sulphide, which can be a detriment to human health directly through physiological effects and indirectly through secondary environmental health effects. Hydrogen sulphide, aromatic compounds, and halogenated compounds posed the greatest risk to human health. These emissions complicated multiple organ systems and increased the chance of death and cancer. This study has the potential to inform decision-makers to enact food waste policies that can prevent the adverse human health effects of food waste emissions. Solutions to reduce food waste emissions have also been explored, such as food diversion programs, emission capture and filter technologies for compost, and creating biofuel. However, few policies have been implemented to reduce food waste emissions, including food redistribution and food waste education programs. Funding more primary research in the entire food waste sector is needed to raise awareness of associated health risks and help decision-makers formulate plans of action.

## Supporting information

S1 Fig Search strategy strings. (PDF)

S2 Fig Full-text screening of identified articles 2021. (PDF)

S3 Fig Full-text screening of identified articles 2022 and 2023. (PDF)

S4 Fig Articles from references. Articles included for data analysis from references of articles included from the search strategy (n = 4).
(PDF)

S5 Fig Critical appraisals of search strategy 2021. (PDF)

S6 Fig Critical appraisals of search strategy 2022. (PDF)

S1 Graphical abstract The effects of gases from food waste on human health: A systematic review. (PDF)
