# Supplementary material for: The effects of gases from food waste on human health: A systematic review
Source: PLoS One. 2024 Mar 27;19(3):e0300801. doi: 10.1371/journal.pone.0300801 (PMC10971579; doi:10.1371/journal.pone.0300801)
Supplement: S1 Fig — (PDF) [file pone.0300801.s001.pdf]

Database	Search String
Web of Science	(food waste (All Fields) and human health (All fields) and gases (All Fields))<br><br>(food waste (All Fields) and human health (All fields) and emissions (All Fields))<br><br>(food waste (All Fields) and human health (All fields) and vapours (All Fields))<br><br>(compost emissions (All Fields) and human health (All fields))
OVID	See image below
EMBASE	See image below
Scopus	((TITLE-ABS-KEY (food AND waste) AND TITLE-ABS-KEY (human AND health) AND TITLE-ABS-KEY (gases))<br><br>((TITLE-ABS-KEY (food AND waste) AND TITLE-ABS-KEY (human AND health) AND TITLE-ABS-KEY (emissions))<br><br>((TITLE-ABS-KEY (food AND waste) AND TITLE-ABS-KEY (human AND health) AND TITLE-ABS-KEY (vapours))<br><br>((TITLE-ABS-KEY (compost AND emissions) AND TITLE-ABS-KEY (human AND health))

Note: No restrictions on date or language

OVID(Medline)

▼ Search History (10) View

	# ▲	Searches	Results	Type	Actions	Annotations
☐	1	Food/	38168	Advanced	Display Results | More ▾	
☐	2	human health.mp.	75198	Advanced	Display Results | More ▾	
☐	3	Gases/ or Greenhouse Gases/ or gases.mp.	67579	Advanced	Display Results | More ▾	
☐	4	1 and 2 and 3	3	Advanced	Display Results | More ▾	
☐	5	Air Pollutants, Occupational/ or Air Pollutants/ or Occupational Exposure/ or vapour.mp. or Gases/	155556	Advanced	Display Results | More ▾	
☐	6	1 and 2 and 5	2	Advanced	Display Results | More ▾	
☐	7	Air Pollution/ or Air Pollutants/	80713	Advanced	Display Results | More ▾	
☐	8	1 and 2 and 7	2	Advanced	Display Results | More ▾	
☐	9	Composting/	2327	Advanced	Display Results | More ▾	
☐	10	2 and 3 and 5 and 9	1	Advanced	Display Results | More ▾	

Embase

☐	1	food waste.mp. or food waste/
☐	2	public health/ or health/ or human health.mp.
☐	3	gases.mp. or gas/
☐	4	1 and 2 and 3
☐	5	composting/ or compost/
☐	6	2 and 3 and 5
