# Supplementary material for: The effects of gases from food waste on human health: A systematic review
Source: PLoS One. 2024 Mar 27;19(3):e0300801. doi: 10.1371/journal.pone.0300801 (PMC10971579; doi:10.1371/journal.pone.0300801)
Supplement: S2 Fig — (PDF) [file pone.0300801.s002.pdf]

Reference information					Instruction	Study design	Context	Outcome				
Reviewer initials	Ref ID	Authors	Title	Year	If the answers to the questions related to all three inclusion criteria (study design, population, outcomes) are all 'yes', the study is eligible for inclusion.<br><br>If the answer to any of the question related the three inclusion criteria (study design, population, outcomes) is 'no', please stop screening and move to the next reference.<br><br>Similarly, for other quesions, if you have doubts or identify issues that need follow-up, give your best answer but then describe additional issues in the comments column.<br><br>You don't need to answer the questions in order and as soon as you answer a question "no" you can stop for that study.	Primary research is to be included for answering thesis question (results section)<br><br>Systematic reviews, commentaries and editorials are to be excluded.<br><br>Is this a primary research study that answers the thesis question: Impact of food waste gases on human health<br><br>1=Yes<br>0=No	Does the study mention all three: 1. food waste, 2. food waste emissions, 3. human health impacts<br><br>Does the study mention all three: 1. food waste, 2. food waste emissions, 3. human health impacts?<br><br>1=Yes<br>0=No	Only interested in food waste emissions that directly or indirectly impact human health, but mentioned in the article<br><br>Does the study mention food waste emissions directly or indirecty related to human health?<br><br>1=Yes<br>0=No	**INCLUDE?**<br>(Autofilled according to your responses to the screening questions)	**Reviwer comment**	**EVANS**	**Consensus**
PR	Schnek, 2019	Schnek, E. C.	Environmental Stewardship in Nursing: Introducing the "WE ACT-*PLEASE*" Framework	2019		0			Exclude	Wrong study design	Exclude	Exclude
PR	Williams, 2019	Williams, P.	Broadening influence on the food supply and environmental sustainability	2019		0			Exclude	Wrong study design	Exclude	Exclude
PR	Conrad, 2020	Conrad, Z.	Food waste, healthy diets, and Environmental Sustainability	2020		0			Exclude	Wrong study design	Exclude	Exclude
PR	Sundin et al., 2021	Sundin, N., Rosell, M., Eriksson, M., Jensen, C., & Bianchi, M.	The impact of exccess food intake- an avoidable environmental burden	2021		1	0		Exclude	Wrong study design	Exclude	Exclude
PR	Vazquez-Rowe et al., 2021	Vazquez-Rowe, I., Ziegler-rodriguez, K., Margallo, M., Kahhat, R., & Aldaco, R.	Climate action and food security: Strategies to reduce GHG emissions from food loss and waste in emerging economies	2021		1	0	0	Exclude	health as in healthy diets	Include	Exclude

PR	Nikkah et al., 2021	Nikkhah, A., Firouzi, S., Dadaei, K., & Van Haute, S.	Measuring circularity in food supply chain using life cycle assessment; Refining oil from olive kernel	2021		1	1	1	Exclude	oil	Include	Exclude
PR	Macpherson et al., 20	Macpherson, C. C., Smith, E., & Rieder, T. N.	Does Health Promotion harm the environment?	2020		0			Exclude	Wrong study design	Exclude	Exclude
PR	Zheng et al., 2020	Zheng, G., Liu, J., Shao, Z., & Chen, T.	Emission characteristics and health risk assessment of VOCs from food waste anaerobic digestion plant: A case study of Suzhou, China	2020		1	1	1	Include		Include	Include
PR	To et al., 2019	To, S., Coughenour, C., & Pharr, J.	The environmental imact and formation of meals from the pilot year of a las vegas convention food rescue program	2019		1	1	1	Include	indirect health	Include	Include
PR	Beckerman et al., 201!	Beckerman, J. P., Blondin, S. A., Richardson, S. A., & Rimm, E. B.	Environmental and economic effects of changing to shelf-stable daairy or soy milk for the breakfast in the classroom program	2019		1	1	0	Exclude	public health mention but doesn't further	Include	Exclude
PR	Jamin et al., 2019	Jamin, N. A., Saleh, S., & Abdul Samad, N. A.	Properties prediction of torrefied municipal solid waste using linear correlation model	2019		1	1	1	Include	indirect health	Exclude	Exclude
PR	Bong et al., 2017	Bong, C. P-C., Goh, R. K. Y., Lim, J-S., Ho, W. S., Lee, C-T., Hashim, H., Abu Mansor, N. N., Ho, C. S., Ramli, A. R., & Takeshi, F.	Towards low carbon society in Iskandar Malaysia: Implementation and feasibility of community organic waste composting	2017		1	1	1	Include	indirect health	Include	Include
PR	Sheikh et al., 2017	Sheikh, S., Baig, M. A., Ali, N., & Khan, N.	Hydrogen sulfide gas poisoning in fish garbage room: A report of a fisherman	2017		1	1	1	Include	direct health, case study	Include	Include
PR	Mao et al., 2006	Mao, I-F., Tsai, C-J., Shen, S-H., Lin, T-F., Chen, W-K., & Chen, M-L.	Critical components of odors in evaluating the performance of food waste composting plants	2006		1	1	1	Include	indirect health	Include	Include

PR	Yatim et al., 2015	Mohd Yatim, S. R., Ku Hamid, K. H., Ismail, K. N., & Abdul Rashid, Z.	Odour profiling from decomposition of local food waste	2015		1	1	1	Include	direct health	Include	Include
PR	Sahar et al., 2015	Sahar, M. A., Nordin, N. J., Mohd Nor, A. T., & Zakaria, M. I.	Weight estimation method in emergency department in malaysia: Is broselow tape (BT) reliable?	2015		1	0		Exclude		Exclude	Exclude
PR	Tsai et al., 2008	Tsai, C-J., Chen, M-L., Ye, A-D., Chou, M-S., Shen, S-H., & Mao, I-F.	The relationship of odor concentration and the critical components emitted from food waste composting plants	2008		1	1	1	Include	indirect health	Include	Include
PR	Sanchez-Monedero et al., 2018	Sanchez-Monedero, M. A., Fernández-Hernández, A., Higashikawa, F. S., & Cayuela, M. L.	Relationships between emitted volatile organic compounds and their concentration in the pile during municipal solid waste composting	2018		1	1	1	Include	indirect health	Exclude	Include
PR	Dias-Ferreira et al., 20	Dias-Ferreira, C., Santos, T., & Oliveira, V.	Hospital food waste and envrionmental and economic indicators- A Portuguese case study	2015		1	0	0	Exclude	good for social implications	Exclude	Exclude
PR	Boliko 2019	Boliko, M. C.	FAO and the situation of food security and nutrition in the world	2019		1	0	0	Exclude	good for intro	Include	Exclude
PR	Nakakubo et al., 2012	Nakakubo, T., Tokai, A., & Ohno, K.	Comparative assessment of technological systems for recycling sludge and food waste at greenhouse gas emissions reduction and phosphorus recovery	2012		1	0	0	Exclude	sludge + FW	Include	Exclude
PR	Drew et al., 2020	Drew, J., Cleghorn, C., Macmillan, A., & Mizdrak, A.	Healthy and climate-friendly eating patterns in the new zealand context	2020		1	0	0	Exclude	dietary health	Include	Exclude
PR	Grizzetti et al., 2013	Grizzetti, B., Pretato, U., Lassaletta, L., Billen, G., & Garnier, J.	The contribution of food waste to global and European nitrogen pollution	2013		0	0	0	Exclude	Wrong study design, good for intro	Include	Exclude

PR	Xian et al., 2021	Xian, C-F., Gong, C., Lu, F., Zhang, L., & Ouyang, Z-Y.	Linking dietary patterns to environmental degradation: The spatiotemporal analysis of rural food nitrogen footprints in China	2021		1	0	0	Exclude	no health, no food waste	Include	Exclude
PR	Flanagan et al., 2021	Flanagan, A., & Priyadarshini, A.	A study of consumer behaviour towards food-waste in Ireland: Attitudes, quantities and global warming potentials	2021		1	0	0	Exclude	no health, good for social aspect	Exclude	Exclude
PR	Barik et al., 2018	Barik, S., Paul, K. K., & Priyadarshi, D.	Utilization of kitchen food waste for biodiesel production	2018		1	0	0	Exclude	no health	Include	Exclude
PR	Klein et al., 2019	Klein, F., Baltensperger, U., Prévot, A. S. H., & El Haddad, I.	Quantification of the impact of cooking processes on indoor concentrations of volatile organic species and primary and secondary organic aerosols	2019		1	0		Exclude	no waste, no health	Exclude	Exclude
PR	Ren et al., 2020	Ren, J., Wang, Z., Niu, D., Huhetaoli, Huang, X., Fan, B., & Li, C.	Isolation and characterization of the novel oil-degrading strain *Kosakonia cowanii* IUMR B67 and expression of the degradation enzyme	2020		1	0	0	Exclude	sludge	Exclude	Exclude
PR	Eustachio Colombo et al., 2020	Eustachio Colombo, P., Patterson, E., Karin Lindroos, A., Parlesak, A, & Schäfer Elinder, L.	Sustainable and acceptable school meals through optimization analysis: an intervention study	2020		1	0	0	Exclude	no health	Include	Exclude
PR	Castner et al., 2017	Castner, E. A., Leach, A. M., Leary, N. Baron, J., Compton, J. E., Galloway, J. N., Hastings, M. G., Kimiecik, J., Lantz-Trissel, J., de la Reguera, E., & Ryals, R.	The nitrogen footprint tool network: a multi-institution program to reduce nitrogen pollution	2017		1	0	0	Exclude	didn't measure food waste	Exclude	Exclude

PR	Mu et al., 2017	Mu, D., Horowitz, N., Casey, M., & Jones, K.	Environmental and economic analysis of an in-vessel food waste composting system at Kean University in the U.S.	2017		1	1	1	Include	Table 7 relevant, indirect health	Include	Include
PR	Fabani et al., 2020	Fabani, M. P., Romań, M. C., Rodriguez, R., & Mazza, G.	Minimization of the adverse environmental effects of discarded onions by avoiding disposal through dehydration and food-use	2020		1	0	0	Exclude	no health	Exclude	Exclude
PR	Makov et al., 2020	Makov, T., Shepon, A., Krones, J., Gupta, C., & Chertow, M.	Social and environmental analysis of food waste abatement via the peer-to-peer charing economy	2020		1	0	0	Exclude	no health, good for policy	Exclude	Exclude
PR	Nordahl et al., 2020	Nordahl, S. L., Devkota, J. P., Amirebrahimi, J., Smith, S. J., Breunig, H. M., Preble, C. V., Satchwell, A. J., Jin, L., Brown, N. J., Kirchstetter, T. W., & Scown, C. D.	Life-cycle greenhouse gas emissions and human health trade-offs of organic waste management strategies	2020		1	1	1	Include	Figure 3 is relevant, health mentioned in context	Include	Include
PR	Conrad et al., 2018	Conrad, Z., Niles, M. T., Neher, D. A., Roy, E. D., Tichenor, N. E., & Jahns, L.	Relationship between food waste, diet quality, and environmental sustainability	2018		1	0		Exclude	no health, good for intro to cropland	Include	Include
PR	Conrad, 2020	Conrad, Z.	Daily cost of consumer food wasted, ineible, and consumed in the United States, 2001-2016	2020		1	0	0	Exclude	good social points	Exclude	Exclude
PR	Woon et al., 2016	Woon, K. S., Lo, I. M. C., Chiu, S. L. H., & Yan, D. Y. S.	Environmental assessment of food waste valorizaton in producing biogas for various types of energy use based on LCA approach	2016		1	1	1	Include	good for solutions	Exclude	Include
PR	White et al., 2011	White, A., Gallegos, D., & Hundloe, T.	The impact of fresh produce specifications on the Australian food and nutrition system: a case study of the north Queensland banana industry	2011		1	0	0	Exclude	didn't measure food waste emissions, good for intro?	Exclude	Exclude
PR	Septiano et al., 2020	Septiano, F., Kemper, J. A., & Northey, G.	Thanks, but no thanks: The influence of gratitude on consumer awareness of food waste	2020		1	0	0	Exclude	social points	Exclude	Exclude
PR	Kanter & Brownlie, 2019	Kanter, D. R., & Brownlie, W. J.	Joint nitrogen and phosphorus management for sustainable development and climate goals	2019		0			Exclude	Wrong study design	Exclude	Exclude
PR	Segrè	Segrè, A.	Food waste prevention to restore food value: Reflections of an agroeconomist	2014						Spanish only		Exclude
PR	Qiao et al., 2020	Qiao, Z., Wei, H. Ma, J., & Xie, B.	Review of the generation of volatile organic compounds during the bio-treatment process of food waste	2020						No full text available		Exclude
												**Total Include: 12**
												**Total Exclude: 28**

Articles from references (below)											
PR	Fleming-Jon	Fleming-Jones, M. E., & Smith, R. E	Volatile organic compounds in foods: A five year study	2003		1	1	1	Include		
PR	Qamaruz-Za	Qamaruz-Zaman, & Milke, M. W.	VFA and ammonia from residential food waste as indicators of odor potential	2012		1	1	1	Include		
PR	Kong et al., :	Kong, X., Liu, J., Ren, L., Song, M., Wang, X., Ni, Z., & Nie, X	Identification and characterization of odorous gas emission from a full-scale food waste anaerobic digestion plant in China	2015		1	1	1	Include		
PR	Mustafa et a	Mustafa, M. F., Liu, Y., Duan, Z., Guo, H., Xu, S., Wang, H., & Lu, W.	Volatile compounds emission and health risk assessment during composting of organic fraction of municipal solid waste	2017		1	1	1	Include		
