# Supplementary material for: The effects of gases from food waste on human health: A systematic review
Source: PLoS One. 2024 Mar 27;19(3):e0300801. doi: 10.1371/journal.pone.0300801 (PMC10971579; doi:10.1371/journal.pone.0300801)
Supplement: S3 Fig — (PDF) [file pone.0300801.s003.pdf]

Reference information					Instruction	Study design	Context	Outcome				
Reviewer initials	Ref ID	Authors	Title	Year	If the answers to the questions related to all three inclusion criteria (study design, population, outcomes) are all 'yes', the study is eligible for inclusion.<br><br>If the answer to any of the question related the three inclusion criteria (study design, population, outcomes) is 'no', please stop screening and move to the next reference.<br><br>Similarly, for other quesions, if you have doubts or identify issues that need follow-up, give your best answer but then describe additional issues in the comments column.<br><br>You don't need to answer the questions in order and as soon as you answer a question "no" you can stop for that study.	Primary research is to be included for answering thesis question (results section)<br><br>Systematic reviews, commentaries and editorials are to be excluded.	Does the study mention all three: 1. food waste, 2. food waste emissions, 3. human health impacts	Only interested in food waste emissions that directly or indirectly impact human health, but mentioned in the article	**INCLUDE?** (Autofilled according to your responses to the screening questions)	**Reviwer comment**		
						Is this a primary research study that answers the thesis question: Impact of food waste gases on human health	Does the study mention all three: 1. food waste, 2. food waste emissions, 3. human health impacts?	Does the study mention food waste emissions directly or indirecty related to human health?				
						1=Yes<br>0=No	1=Yes<br>0=No	1=Yes<br>0=No			EVANS	Consensus
PR	Li et al., 2020	Li H, Qi G, Liu X, Ren L, Zhao Y, Sun Y.	Emission Characteristics and Health Risk Assessment of Odorous Pollutants from Organic Fraction of Municipal Solid Waste Compost in Summer	2020		NA	NA	NA	Exclude	Article in Chinese	Exclude	Exclude
PR	Pankhurst et al., 2021	L. J. Pankhurst, L. J. Deacon, J. Liu, G. H. Drew, E. T. Hayes, S. Jackson, P. J. Longhurst, J. W. S. Longhurst, S. J. T. Pollard & S. F. Tyrrel	Mirobial an endotoxin emission from composting facilities: characterisation of release and dispersal patterns	2021		1	1	1	Include	Endotoxins = aers	Include	Include
PR	Pascale et al., 2021	Erica Pascale, Elena Franchitti, Chiara Caredda , Stefania Fornasero, Giulia Carletto, Biancamaria Pietrangeli, Francesco Valentino, Paolo Pavan, Giorgio Gilli, Elisa Anedda, Deborah Traversi	Bioaerosol emissions during organic waste treatment for biopolymer production: A case study	2021		1	0	0	Exclude		Exlcude	Exclude

PR	Behrooznia et al., 2020	Comparative life cycle environmental impacts of two scenarios for managing an organic fraction of municipal solid waste in Rasht-Iran	Comparative life cycle environmental impacts of two scenarios for managing an organic fraction of municipal solid waste in Rasht-Iran	2020			1	0	0	Exclude	No direct emissions measured	Exlcude	Exclude
PR	Thiel et al., 2021	Cassandra L. Thiel, Si Woon Park, Aviva A. Musicus, Jenna Agins, Jocelyn Gan, Jeffrey Held, Amy Horrocks, Marie A. Bragg	Waste generation and carbon emissions of a hospital kitchen in the US: potential for waste diversion and carbon reductions	2021			1	0	0	Exclude	Mixed compost, no emissions measured	Exlcude	Exclude
PR	Daniel-Gromke et al., 2015	Digestion of bio-waste - GHG emissions and mitigation potential	Digestion of bio-waste- GHG emissions and mitigation potential	2015			1	0	0	Exclude	plant waste	Exlcude	Exclude
PR	Li et al., 2015	Hua Li, Vilas Nitivattananon, & Peng Li	Municipal solid waste mangement health risk assessment from air emissions for China by applying life cycle analysis	2015			1	0	0	Exclude	plant compost, MSW	Exlcude	Exclude
PR	Fischer et al., 1998	G. Fischer, R. Schwalbe, R. Ostrowski, & W. Dott	Airborne fungi and their secondary metabolites in working places in a compost facility	1998			1	1	1	Include		Include	Include
PR	Aatamila et al., 2011	Marjaleena Aatamila, PiaK.Verkasalo, MaaritJ.Korhonen, AnnaLiisa Suominen, Maija-Riitta Hirvonen, MarjaK.Viluksela, AinoNevalainen	Odour annoyance and physical symptoms among residents living near waste treatment centres	2011			1	0	0	Exclude		Exclude	Exclude
PR	Muller et al., 2003	Thomas Miiller, Ralf Thiflen, Silvia Braun, Wolfgang Dott, & Guido Fischer	(M)VOC and composting facilities Part 1: (M)VOC Emissions from municipal biowatse and plant refuse	2003			1	0	0	Exclude	plant waste	Exlcude	Exclude

PR	Grzesik et al., 2014	Grzesik, K., Kozakiewicz, R, & Bieda, B.	Life cycle assessment for landfilling, incineration and mechanical-biological treatment of residual waste for Krakow city (Poland)	2014		1	0	0	Exclude	not all food waste	Exclude	Exclude
PR	Ferguson et al., 2021	Robert M.W. Ferguson, Charlotte E.E. Neath, Zaheer A. Nasir, Sonia Garcia-Alcega, Sean Tyrrel, Frederic Coulon, Alex J. Dumbrell, Ian Colbeck, Corinne Whitby	Size fractionation of bioaerosol emissions from green-waste composting	2021		1	1	1	Include	green waste	Include	Include
PR	Haas et l., 1999	Haas D.U., Reinthaler F.F., Wust G., Skofitsch G., Groth I., Degenkolb T., Schumann P., Marth E.	Emission of thermophilic actinomycetes in composting facilities, their immediate surroundings and in an urban area	1999		NA	NA	NA	Exclude	No full-text available	Exclude	Exclude
PR	Weligama Thuppahige e	Rasangika Thathsaranee Weligama Thuppahige, Shabbir H. Gheewala, Sandhya Babel	Environmental impact of organic fraction of municipal solid waste treatment by composting in Sri Lanka	2021		1	1	0	Exclude	emission data not extractable	Exclude	Exclude
PR	Tolvanen et al., 1998	Tolvanen, O. K., Hanninen, K. I., Veijanen, A., & Villberg K.	Occupational hygiene in biowaste composting	1998		1	1	1	Include		Include	Include
PR	Douglas et al., 2016	P. Douglas, S.F. Tyrrel, R.P. Kinnersley, M. Whelan, P.J. Longhurst, K. Walsh, S.J.T. Pollard, G.H. Drew	Sensitivity of predicted bioaerosol exposure from open windrow composting facilities to ADMS dispersion model parameters	2016		1	0	0	Exclude	no gases measured	Exclude	Exclude
PR	Velusami et al., 2013	Velusami, B., Curran, T.P., Grogan, H.M.	Hydrogen sulfide gas emissions in the human-occupied zone during disturbance and removal of stored spent mushroom compost	2013		1	0	0	Exclude	spent mushroom compost include manure, straw, etc.	Exclude	Exclude
PR	Koeing et al., 2005	Richard T. Koenig, F. Dean Miner, Jr., Bruce E. Miller, & John D. Harrison	Variability of Atmospheric Ammonia In High-Rise, Caged Layer Composting	2005		1	0	0	Exclude	manure in compost	Exclude	Exclude
PR	Deacon et al., 2009	Lewis Deacon, Louise Pankhurst, Jian Liu, Gillian H Drew, Enda T Hayes, Simon Jackson, James Longhurst, Philip Longhurst, Simon Pollard, & Sean Tyrrel	Endotoxin emissions from commercial composting activities	2009		1	1	1	Include		Include	Include
PR	Mbareche et al., 2017	Hamza Mbareche, Marc Veillette, Laetitia Bonifait, Marie-Eve Dubuis, Yves Benard, Geneviève Marchand, Guillaume J. Bilodeau, & Caroline Duchaine	A next generation sequencing approach with a suitable bioinformatics workflow to study fungal diversity in bioaerosols released from two different types of composting plants	2017		1	1	1	Exclude	no raw data to extract, no premission from authors	Include	Exclude
PR	Vilavert et al., 2009	Lolita Vilavert, Martí Nadal, Isabel Inza, María J. Figueras, José L. Domingo	Baseline levels of bioaerosols and volatile organic compounds around a municipal waste incinerator prior to the construction	2009		1	0	0	Exclude	MSW	Exclude	Exclude

PR	Williams et al., 2017	Ben Williams, Enda Hayes, Zaheer Nasir, Catherine Rolph, Simon Jackson, Shagun Khera, Alan Bennett, Toni Gladding, Gillian Drew, & Sean Tyrrel	The challenges, uncertainties and opportunities of bioaerosol dispersion modelling from open composting facilities	2017		1	0	0	Exclude	a model	Exclude	Exclude
PR	Reyes et al., 2020	J. Reyesa, M.C. Gutiérreza, M. Toledoa, L. Verab, L. Sánchezc, J.A. Silesa, & M.A. Martina	Environmental performance of an industrial biofilter: Relationship between photochemical oxidation and odorous impacts	2020		1	1	1	Include	odours related to human health, thresholds extractable	Include	Include
PR	Schiavon et al., 2017	Marco Schiavon, Luca Matteo Martini, Cesare Corr, Marco Scapinello, Graziano Coller, Paolo Tosi, Marco Ragazzi	Characterisation of volatile organic compounds (VOCs) released by the composting of different waste matrices	2017		1	1	1	Include		Include	Include
Articles from references (below)												
PR	Wikandari et al., 2013	Wikandari, R, Gudipudi, S, Pandiyan, I, Millati, R, & Taherzadeh, MJ	Inhibitory effects of fruit flavors on methane production during anaerobic digestion.	2013		1	1	1	Include	Food waste gases from fruit	Include	Include
PR	Winniczuk et al., 1997	Winniczuk, PP, & Parish, ME	Minimum inhibitory concentrations of antimicrobials against micro-organisms related to citrus juice. Food Microbiology	1997		1	1	1	Include	Food waste gases from fruit juice	Include	Include
PR	Parales, 2010	Parales, RE	Hydrocarbon degradation by betaproteobacteria	2010		1	1	1	Exclude	Degradation procss of food waste gases	Include	Exclude
PR	Huang et al., 2018	Huang, C-C, Zeng, Y-H, Luo, X-J, Tang, B, Liu, Y-E, Ren, Z-H, et al	Level changes and human dietary exposure assessment of halogenated flame retardant levels in free-rage chicken eggs: A case study of a former e-waste recycling site, South China.	2018		1	1	1	Include	Chicken egg waste and impact on human health	Include	Include
PR	Cowart et al., 1997	Cowart, BJ, Young, IM, Feldman, RS, & Lowry, LD	Clinical disorders of smell and taste. Occupational Medicine	1997		1	1	1	Exclude	Odours and effcts on olfactory system and oral health	Exclude	Exclude
PR	Miwa et al., 2001	Miwa, T, Furukawa, M, Tsukatani, T, Costanzo, RM, DiNardo, LJ, & Reiter, ER	Impact of olfactory impairment on quality of life and disability	2001		1	1	1	Exclude	Odours and effcts on the olfatory system	Include	Exclude
PR	Xing et al., 2016	Xing, Y-F, Xu, Y-H, Shi, M-H, & Lian, Y-X.	The impact of PM2.5 on the human respiratory system. Journal of Thoracic Disease	2016		1	1	1	Exclude	Effects of PM2.5 on the human respiratory system	Excude	Exclude
PR	Swan et al., 2003	Swan, JRM., Kelsey, A, Crook, B, & Gilbert, EJ	[illegible] environmental exposure to bioaerosols from composts and potential health effect- a critical review of published data.	2003		1	1	1	Exclude	Bioaerosols from compost, systematic review	Exclude	Exclude
PR	Douwes et al., 2003	Douwes, J, Thorne, P, Pearce, N, & Heederik, B.	Bioaerosol health effects and exposure assessment: progress and prospects.	2003		1	0	1	Exclude	Human effects of bioaerosols	Exclude	Exclude
PR	Flaherty et al., 1984	Flaherty, DK., Deck, FH, Cooper, J, Bishop, K, Winzenburger, PA, Smith, LR, et al	Bacterial endotoxin isolated from a water spray air humidification system as a putative agent of occupation-related lung disease	1984		1	0	1	Exclude	Effect of endotoxins	Exclude	Exclude
PR	Cariou et al., 2016	Cariou, S, Fanlo, J-L, Stitou, Y, Buty, D, Samani, D, & Akiki, R.	Application of ODEMS (odorant dispersion and emissions monitoring system) to measure odorous emissions from composting plant.	2016		1	1	1	Exclude	Odours released from gases, effects on human health, no data extractable	Include	Exclude

Reference information					Instruction	Study design	Context	Outcome				
Reviewer initials	Ref ID	Authors	Title	Year	If the answers to the questions related to all three inclusion criteria (study design, population, outcomes) are all 'yes', the study is eligible for inclusion.<br><br>If the answer to any of the question related the three inclusion criteria (study design, population, outcomes) is 'no', please stop screening and move to the next reference.<br><br>Similarly, for other quesions, if you have doubts or identify issues that need follow-up, give your best answer but then describe additional issues in the comments column.<br><br>You don't need to answer the questions in order and as soon as you answer a question "no" you can stop for that study.	Primary research is to be included for answering thesis question (results section)<br><br>Systematic reviews, commentaries and editorials are to be excluded.<br><br>Is this a primary research study that answers the thesis question: Impact of food waste gases on human health<br><br>1=Yes<br>0=No	Does the study mention all three 1. food waste, 2. food waste emissions, 3. human health impacts<br><br>Does the study mention all three: 1. food waste, 2. food waste emissions, 3. human health impacts?<br><br>1=Yes<br>0=No	Only interested in food waste emissions that directly or indirectly impact human health, but mentioned in the article<br><br>Does the study mention food waste emissions directly or indirecty related to human health?<br><br>1=Yes<br>0=No	INCLUDE?<br>(Autofilled according to your responses to the screening questions)	Reviwer comment	EVANS	Consensus
PR	Cheng & Leong	Cheng & Leong	Data-driven decarbonisation pathways for reducing life cycle GHG emissions from food waste in the hspitalityan food service sectors	2023		1	1	1		Climate change impacts human health indirectly	Exclude	Exclude
PR	Berardy et al., 2022	Andrew Berardy, Brianna Egan, Natasha Birchfield, Joan Sabaté, & Heidi Lynch	Comparison of plate waste between vegetarian and meat-consuming meals in a hospital setting: Environmental and nutritional considerations	2022		1	0		Exclude, no exrtactabe info		Exclude	Exclude
PR	Thuppahige et al., 2022	Thuppahige et al.	Rasangika Thathsaranee Weligama Thuppahige, Shabbir H Gheewala, Sandhya Babel	2022		1	1	1	Include		Exclude	Exclude
PR	Zhang et al., 2022	Liangmao Zhang, Binghan Wang, Zijiang Wang, Kaiyi Li, Ru Fang, Yinglong Su, Dong Wu, Bing Xie	Spatiotemporal footprints of odor compounds in megacity's food waste streams and policy implication	2022		1	1	0	Exlcude, no extractable info		Exclude	Exclude
PR	Thiel et al., 2021	Thiel et al.	Waste generation and carbon emissions of a hospital kitchen in the US: Potential for waste diversion and carbon reductions	2021		1	0		Exclude		Exclude	Exclude
PR	Doney et al., 2021	Brent Doney, Laura Kurth, Girija Syamlal	Chronic bronchitis and emphysema among workers exposed to dust, vapors, or fumes by industry and occupation	2021		1	0		Exclude		Include	Exclude
PR	Nguyen et al., 2023	Minh Ky Nguyen, Chitsan Lin, Hong Giang Hoang, Xuan Thanh Bui, Huu Hao Ngo, Van Giang Le, Huu-Tuan Trani	Investigation of biochar amendments on odor reduction and their characteristics during food waste co-composting	2023		1	0		Exclude		Exclude	Exclude
PR	Sundin et al., 2021	Niina Sundin, Magdalena Rosell, Mattias Eriksson, Carl Jensen, Marta Bianchi	The climate impact of excess food intake - An avoidable environmental burden	2021		1	0		Exclude	Doesn't specify which gases	Exclude	Exclude
