# Supplementary material for: The effects of gases from food waste on human health: A systematic review
Source: PLoS One. 2024 Mar 27;19(3):e0300801. doi: 10.1371/journal.pone.0300801 (PMC10971579; doi:10.1371/journal.pone.0300801)
Supplement: S4 Fig — Articles included for data analysis from references of articles included from the search strategy (n = 4). (PDF) [file pone.0300801.s004.pdf]

| **Lead Author, Year** | **Title** |
|---|---|
| Fleming-Jones et al., 2003 | Volatile organic compounds in foods: A five-year study |
| Kong et al., 2015 | Identification and characterization of odorous gas emission from a full-scale food waste anaerobic digestion plant in China |
| Mustafa et al., 2017 | Volatile compounds emission and health risk assessment during composting of organic fraction of municipal solid waste |
| Qamaruz-Zaman & Milke, 2012 | VFA and ammonia from residential food waste as indicators of odour potential |
| Wikandari et al., 2013 | Inhibitory effects of fruit flavors on methane production during anaerobic digestion. |
| Winniczuk et al., 1997 | Minimum inhibitory concentrations of antimicrobials against micro-organisms related to citrus juice. |
| Huang et al., 2018 | Level changes and human dietary exposure assessment of halogenated flame retardant levels in free-rage chicken eggs: A case study of a former e-waste recycling site, South China. |
