# Supplementary material for: The effects of gases from food waste on human health: A systematic review
Source: PLoS One. 2024 Mar 27;19(3):e0300801. doi: 10.1371/journal.pone.0300801 (PMC10971579; doi:10.1371/journal.pone.0300801)
Supplement: S5 Fig — (PDF) [file pone.0300801.s005.pdf]

Qualitative Studies

Author	**Study purpose:** was the purpose of the study	**Literature:** was relevant background literature	**Design:** Was the research experimental or	**Sampling Criteria:** Is the sample size	**Outcomes:** were the outcome measures	Were the outcome measures valid? Yes, no,	Specify outcome measures	**Experiment:** are the experimental methods	Are the variables independently tested? Yes	**Results:** Is the data analysis congruent with	**Ethical approval:** Did the study receive ethical	**Conclusions:** were the study conclusions	List any limitations and biases
Boliko, 2019	yes	yes	N/A, report	N/A	no	N/A	N/A	N/A	not adressed	N/A	N/A	N/A	report
Bong, 2017	yes	yes	experimental	N/A	yes	yes	GHGs from 3 senarios	yes	yes	yes	no, community engagement	yes	None
Conrad, 2018	yes	yes	Secondary	yes	yes	yes	food waste	N/A	yes	yes	N/A	yes	low reporting, outdated
Mao, 2006	yes	yes	experimental	N/A	yes	yes	odours	yes	yes	yes	N/A	yes	None
Mu, 2017	yes	yes	experimental	N/A	yes	yes	compost emissions	yes	yes	yes	N/A	yes	None
Nikkhah, 2021	yes	yes	experimental	N/A	yes	yes	emissions, human health	yes	yes	yes	N/A	yes	only looked at kernel oil
Nordahl, 2020	yes	yes	experimental	N/A	yes	yes	kgCO2 per Tonne waste	yes	yes	yes	N/A	yes	None
Sanchez-Monedero, 2018	yes	yes	experimental	N/A	yes	yes	CO2 and CH4 emissions	yes	yes, organic waste	yes	N/A	yes	None
To, 2019	yes	yes	experimental	N/A	yes	yes	CO2, CH4, NO2 emissions	yes	yes, food waste	yes	N/A	yes	None
Tsai, 2008	yes	yes	experimental	N/A	yes	yes	VOCs from food waste	yes	yes	yes	N/A	yes	None
Woon, 2016	yes	yes	experimental	N/A	yes	yes	food waste emissions	yes	yes	yes	N/A	yes	None
Yatim, 2015	yes	no	experimental	N/A	yes	yes	VOCs from food waste	yes	yes	yes	N/A	yes	Residential dietary bias
Zheng, 2020	yes	yes	experimental	N/A	yes	yes	odours	yes	yes	yes	N/A	yes	None
Articles from references													
Fleming-Jones & Smith, 2003	yes	yes	experimental	yes	yes	yes	food waste emissions	yes	yes	yes	N/A	yes	None
Qamaruz-Zaman & Milkem 2012	yes	yes	experimental	yes	yes	yes	odour rates	yes	yes	yes	NR	yes	not sure if it's ethical
Kong et al., 2015	yes	yes	experimental	yes	yes	yes	VOCs food waste	yes	yes	yes	N/A	yes	None
Mustafa et al., 2017	yes	yes	experimental	yes	yes	yes	food waste emissions	yes	yes	yes	N/A	yes	None

Case Study

**Author, year**	**Note: Answers to each question can be yes, no, unclear, not applicable**	Were patient's demographic characteristics clearly described?	Was the patient's history clearly described and presented as a timeline?	Was the current clinical condition of the patient on presentation clearly described?	Were diagnostic tests or assessment methods and the results clearly described?	Was the intervention(s) or treatment procedure(s) clearly described?	Was the post-interventionclinical condition clearly described?	Were adverse events (harms) or unanticipated events identified and described?	Dose the case report provide takeaway lessons?
Sheikh, 2017		No	No	Yes	Yes	Yes	No	No	Yes
