# Supplementary material for: The effects of gases from food waste on human health: A systematic review
Source: PLoS One. 2024 Mar 27;19(3):e0300801. doi: 10.1371/journal.pone.0300801 (PMC10971579; doi:10.1371/journal.pone.0300801)
Supplement: S6 Fig — (PDF) [file pone.0300801.s006.pdf]

Author	**Study purpose:** was the purpose of the study stated clearly? Yes or No	**Literature**: was relevant background literature reviewed? Yes or No	**Design**: Was the research experimental or secondary? Yes or No and Describe	**Sampling Criteria**: Is the sample size representative? Is the sampling criteria justified?	**Outcomes**: were the outcome measures reliable? (Yes, No, not adressed)	Were the outcome measures valid? Yes, no, not adressed	Specify outcome measures	**Experiment:** are the experimental methods clear? Yes or no	Are the variables independently tested? Yes or No	**Results**: Is the data analysis congruent with the methodology? Yes or No	**Ethical approval**: Did the study receive ethical apporaval? Yes, No, N/A	**Conclusions:** were the study conclusions appropriate given the study methods and	List any limitations and biases
Pankhurst, 2021	yes	yes	yes	yes	yes	yes	Actinomycetes, endotoxin, gram negative-bacteria emissions	yes	yes	yes	not applicable	yes	None
Fischer, 1998	yes	yes	yes	yes	yes	yes	Airborne fungi species emitted from a compost facility	yes	yes	yes	not applicable	yes	One compost unit sampled
Ferguson, 2021	yes	yes	yes	yes	yes	yes	Bioaerosol sampling, identification from compost sites	yes	yes	yes	not applicable	yes	Primer choice, metabarcoding, metagenomics used
Tolvanen, 1998	yes	yes	yes	yes	yes	yes	Volatile organic compounds, airborne microbe, endotoxins emissions and odours from compost	yes	yes	yes	not applicable	yes	Odour thresholds may vary from country to country
Deacon, 2009	yes	yes	yes	yes	yes	yes	Endotoxins and bioaersol emissions from compost	yes	yes	yes	not applicable	yes	None
Mbareche, 2017	yes	yes	yes	yes	yes	yes	Fungal bioaerosols from organic compost	yes	yes	yes	not applicable	yes	Raw data is not reported
Reyes, 2020	yes	yes	yes	yes	yes	yes	Chemical gas emissions and odours from organic compost	yes	yes	yes	not applicable	yes	Raw data is not reported
Schiavon, 2017	yes	yes	yes	yes	yes	yes	Volatile organic compounds and odours from compost	yes	yes	yes	not applicable	yes	Raw data is not reported
