# Supplementary material for: The effects of gases from food waste on human health: A systematic review
Source: PLoS One. 2024 Mar 27;19(3):e0300801. doi: 10.1371/journal.pone.0300801 (PMC10971579; doi:10.1371/journal.pone.0300801)

The effects of gases from food waste on human health: A systematic review

Household food waste emissions are poorly understood in literature, yet the emissions impact human health on a spectrum of severity.

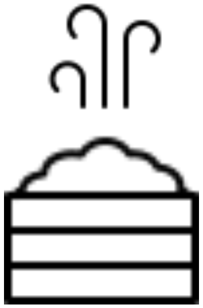

Organic household food waste emits volatile organic carbons, carbon dioxide, carbon monoxide, bioaerosols, and endotoxins. Such emissions directly impact human health, ranging from respiratory system complications and olfactory cell changes to loss of consciousness and death. Indirect impacts on human health range from global warming and smog to acid rain.

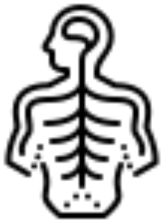

The human health impacts of food waste emissions can be avoided by implementing policies. Such initiatives could include food diversion programs, gas capture and filter technologies, and biofuel generation.

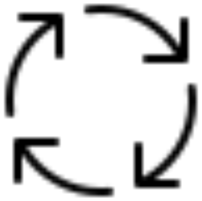

Supplement: S1 Graphical abstract — (PDF) [file pone.0300801.s007.pdf]
